# From Controlled to Automatic Processes and Back Again: The Role of Contextual Features

**DOI:** 10.5964/ejop.v15i4.1746

**Published:** 2019-12-19

**Authors:** Rosa Angela Fabio, Tindara Caprì, Martina Romano

**Affiliations:** a Department of Clinical and Experimental Medicine, University of Messina, Messina, Italy; Webster University Geneva, Geneva, Switzerland

**Keywords:** automaticity, context-specificity, controlled and automatic processes, cognition

## Abstract

In cognitive psychology, classical approaches categorize automatic and controlled processes from a dichotomous point of view. Automatic processes are believed to be rigid, whereas controlled processes are thought to be flexible. New theories have softened this dichotomous view. The aim of the present study is to examine the possibility of implementing flexibility in automatic processing through reliance on contextual features. One hundred and twenty subjects (mean age 22.4, SD = 4.2), 60 male and 60 female, participated in this study. An automatic sequence task (with and without contextual features) was used to test flexibility in automatic processing. Results showed that the use of contextual cues can increase flexibility in automatic processes. The results are discussed in light of new theories on softened automaticity.

The first distinction between automatic and controlled processes was introduced by Shiffrin and Schneider (1977; see also Schneider & Chein, 2003) with the dual-process theory. This theory suggests that there are two distinctive cognitive processes, namely, controlled and automatic processes (also called controlled cognition and automatic cognition, respectively) that play roles in predicting behavior. Automatic processing is fast, effortless, autonomous, stereotypic, unavailable to conscious awareness and fairly error-free. It can be accomplished simultaneously with other cognitive processes without interference, it is not limited by attentional capacity and it can be unconscious or involuntary (Cuzzocrea, Grasso, & Nucita, 2014; Fabio, 2009, 2017; Fabio & Antonietti, 2012; Fabio & Caprì, 2015, 2017, 2019; Fabio et al., 2018a; Grasso, Cuzzocrea, & Nucita, 2014; Martino, Caprì, Castriciano, & Fabio, 2017; Mohammadhasani, Fabio, Fardanesh, & Hatami, 2015; Mohammadhasani, Fardanesh, Hatami, Mozayani, & Fabio, 2018; Moors & De Houwer, 2006). Most commonly, automatic cognitions are depicted as unconscious mental associations between concepts and valences in an associative network or in habitual responses that can generate quick, spontaneous behavioural tendencies without intention (Xu, Li, Ding, & Lu, 2014). In contrast, controlled processing is effortful, slow, and prone to errors, but at the same time, flexible and useful to deal with novel situations (Fabio, 2009; Fabio & Urso, 2014; Moors & De Houwer, 2006; Nucita et al. 2013). Furthermore, in controlled processing, people carry out deliberate behaviors by retrieving information from memory with effort which is particularly important as it support behaviours that achieve goals and ultimately promote survival (Xu et al., 2014).

According to classical theories of cognitive control and automaticity, automaticity has been considered as an all-or-none phenomenon, that is, a process is either automatic or controlled. Controlled processes were considered exclusive to the domain of conscious cognition, and automatic processes were thought to be in the domain of unconscious cognition (Posner & Snyder, 1975; Shiffrin & Schneider, 1977). This classical view implies that a behavioral or neurophysiological effect has to be context-independent to induce a “truly automatic” process (Abrahamse & Verwey 2008; Pessoa, Kastner, & Ungerleider, 2003). However, it is difficult to identify processes that actually meet the classical criteria for automaticity as task demands frequently modulate behavioral and neurophysiological effects (Kiefer, 2012; Moors & DeHouwer, 2006). Behavioural studies also suggest a set of phenomena associated with automaticity that indicate a continuous process rather than an all-or-none process: gradual development with practice; concomitant improvements in speed (and a reduction of variance); reduced reliance on, but not complete autonomy from, the effects of attention, the relative nature of interference effects and the interacting influences of stimulus information and attentional allocation on responding (Cohen, Servan-Schereiber, Mellon, & McClelland, 1992). Moreover, even if most daily tasks are performed almost automatically, it is sometimes necessary to alter a routine if something changes in the environment and routine behavior becomes inappropriate. In some “real-world” situations the habituation/automatization processes may lead to disadaptive behaviour, for example, in learning settings, when a student automatize the wrong procedure to solve a problem may be useful to contrast such wrong automatization; moreover, in clinical settings, patients may need to stop automatic negative thoughts and to switch to positive ones to reach a better adaptation to the external world.

This behavioural switching can occur either retroactively based on error feedback or proactively by detecting a contextual cue (Hikosaka & Isoda, 2010), so, it requires a shift from automatic to controlled processes and back again. Summarizing, such evidence suggests that automatic processing can be flexible and context-dependent.

Unlike classical theories, refined theories of automaticity and unconscious processing allow for more flexibility and adaptability of unconscious automatic processing (Fabio, Gangemi, Caprì, Budden, & Falzone, 2018b; Fabio, Caprì, Nucita, Iannizzotto, & Mohammadhasani, 2018; Fabio, Magaudda, Caprì, Towey, & Martino, 2018c; Kiefer, 2007, 2012; Kiefer & Martens, 2010; Moors & DeHouwer, 2006; Naccache, Blandin, & Dehaene, 2002; Neumann, 1990). These theories postulate that unconscious or automatic processing, in general, depends on a configuration of the cognitive system regarding attention and task sets. In his theory of direct parameter specification (DPS), Neumann (1990) proposes that unconscious information will only be processed and will influence the motor response to a target stimulus to the extent that it matches current intentions. Similarly, the global workspace model of consciousness by Dehaene and Naccache (2001) explicitly assumes that unconscious processes are susceptible to attentional amplification. By contrast to classical theories, refined theories propose that executive control factors such as attention, intentions, and task sets orchestrate the unconscious processing streams toward greater optimization of task performance. Summarizing, recent evidence shows that automatic processes can develop gradually with practice and, furthermore, that they may depend on the context in which they are evaluated.

In line with aforementioned new theoretical perspectives, a key issue for researchers is to understand whether and how automatic processes can be flexible and adaptive in spite of their apparent rigidity. One potential answer to this question is that automaticity gains flexibility through sensitivity to contextual cues (D’Angelo, Milliken, Jimenez, & Lupinez, 2013). Context refers to those perceptual features of the task setting that are not formally required for successful task performance, yet which may influence performance with practice based on contingencies with task-relevant information. Indeed, context-dependent learning and related concepts such as procedural reinstatement or specified learning patterns are typically related to the notion that context-features become associated with the task during acquisition and subsequently enhance performance by acting as a cue for memory retrieval processes (Ruitenberg, Abrahamse, De Kleine, & Verwey, 2012a).

D’Angelo, Jiménez, Milliken, and Lupiáñez (2012; see also D’Angelo et al., 2013) investigated the role of contextual factors in the expression of automaticity in an implicit learning task. In three experiments, the authors showed that participants can learn two complementary sequences implicitly that are associated with distinct contexts, and that when the two contexts are randomly intermixed automaticity depends on the distinctiveness of the two contexts. The results demonstrated that context-specific characteristics can control the acquisition and expression of implicit sequence knowledge. Moreover, in a recent study, Ruitenberg, Verwey, and Abrahamse (2015) examined whether changes in the perceptual context can have a differential impact across distinct processing phases (preparation vs. execution of a motor chunk) within an ongoing movement sequence. Their results confirmed context-dependence in sequential action. Anyway, re-examining the role of context- cue in implicit sequence learning, D’Angelo, Milliken, Jiménez, and Lupiáñez (2014) conducted two experiments but did not confirm their previous results. They confirmed that two complementary sequences can be learned on alternating blocks during the training phase, but the authors do not find a facilitation effect due to the context. Therefore, the literature is unclear about the conditions under which context can implement flexibility in automatic processing of learned sequences. More works are needed to clarify how automatic processes can be flexible through reliance on contextual features (D’Angelo, 2012, 2013).

In light of this knowledge gap, the main focus of the current paper is to analyze how the learned task could become more flexible through the inclusion of context-specific features. In this study, learning of a sequence task was used to test automatic processing. According to previous studies (Caprì, Gugliandolo, Iannizzotto, Nucita, & Fabio, 2019; D’Angelo, 2012, 2013, 2014; Fabio, 2017; Fabio, Caprì, Iannizzotto, Nucita, & Mohammadhasani, 2019; Fabio, Iannizzotto, Nucita, & Caprì, 2019; Logan, 1988, 1989; Ruitenberg et al., 2012a, 2015; Ruitenberg, De Kleine, Van der Lubbe, Verwey, & Abrahamse, 2012b) automaticity can develop from previous experiences and can be revealed by the gradual increment of processing speed.

The specific aim was to demonstrate that participants can increase the processing speed (automatization) of two sequence tasks, and that this increment can be tied to contextual features associated with the two tasks. The first goal of this study was to induce an automatization process of two sequence tasks. The second aim was to examine whether the contextual features influenced flexibility on the learned sequences.

The role of contextual features was evaluated through the manipulation of the distinctiveness of contextual information. In the condition with contextual cue, we helped the subject to recognize the target by providing two different contexts for each different target. In the condition without contextual cue, the context was always the same.

Summarizing, we hypothesized that: 1) when the subjects identified the same target stimulus during many trials (first sequence), speed of execution and accuracy in the identification of target would increase (showing gradual automatization process); 2) after the first sequence automatization, the target changed in two different ways: with and without contextual distinctiveness (cue), therefore we awaited that subjects in the condition without contextual-cue would show a loss of processing speed; whereas in the condition with contextual-cue, the subjects might not show this loss; 3) successively a second sequence with the second target for many trials was presented and when the two targets were intermixed (and the controlled processes were necessary to identify the two different targets), we awaited that the loss of processing speed and accuracy should be higher in the condition without contextual-cue.

## Method

### Participants

One hundred and twenty subjects between 18 and 30 years (mean age 22.4, *SD* = 4.2), 60 male and 60 female, participated in this study. They were individually asked to indicate their level and type of education: 62% of the subjects had been or were about to achieve a secondary diploma in the scientific or humanistic field, 38% had a bachelor's degree or worked. All participants had normal or corrected to normal vision. Participants were recruited through an article in the local newspaper, a university e-newsletter, and networks of acquaintance. Each subject voluntarily agreed to participate in this research study. After providing informed consent, they were tested individually.

### Task

The experimental task was the Clock Test Sequences (Moron, 1997), a visual-spatial attention test used in previous works to investigate the automaticity (Szymura, Slabosz, & Orzechowski, 2001; Fabio, Castriciano, & Rondanini, 2015; Valle, Massaro, Castelli, & Marchetti, 2015). The Clock test consisted of 400 visual stimuli: 40 targets and 40 distractors. The target stimulus was a black and white clock-face showing 04:00 or 05:00 hours on a white background. The distractor stimulus was another clock-face showing different hours. The stimuli were presented on a white sheet. The participants were required to mark only the target stimulus (the clock in the header line on the sheet) mixed with others (distractor stimuli), as fast as they were able.

### Procedure

Subjects were seated in a well-lit, quiet room. The Clock Test Sequence was presented seven consecutive times for 2 minutes each. In the first three trials, the target clock-face was the one indicating 4 o’clock. These three trials were also called “first learned sequence.” From the fourth to the sixth trial, the target clock-face was 5 o’clock. This sequence was called “second learned sequence.” In the seventh trial, the target clock-faces were both 4 and 5 o’clock that were randomly intermixed. This trial was called “intermixed task.”

Following this stimulus sequence, we wanted to induce an automatization process of two target clock-faces and we also evaluated flexibility in two ways: with the shift of the target clock-face after the first target was automatized (from 3^rd^ to 4^th^ trial); and with an intermixed presentation of the first and second target (during the 7^th^ trial). The loss of processing speed, between the 3^rd^ and 4^th^ trial, and after, between the 6^th^ and 7^th^ trial, was considered an index of the rigidity/flexibility continuous process.

As described in the “task” section, the participants were asked to mark the stimulus targets as quickly as possible, from left to right, following the instructions for searching for targets. The subjects were presented with seven sheets of paper with a different ordering of clock-faces throughout all the trials. Test duration was 2 minutes for each sheet.

Since the aim was to examine whether contextual features influenced flexibility on the learned sequences task, the Clock Test Sequence was presented in two conditions: “with and without contextual cue.” The term context refers to features that can help the participants in distinguishing between different sources of information and in recognizing the two target clock-faces. In this experiment, we manipulated the distinctiveness of contextual information in the two conditions, providing two different contextual cues for the two targets. Precisely, the contextual cue was the shape of clock-faces depicted on the sheet: a circle for the clocks showing 04:00 and a square for the clocks showing 05:00, respectively.

**Figure 1:**
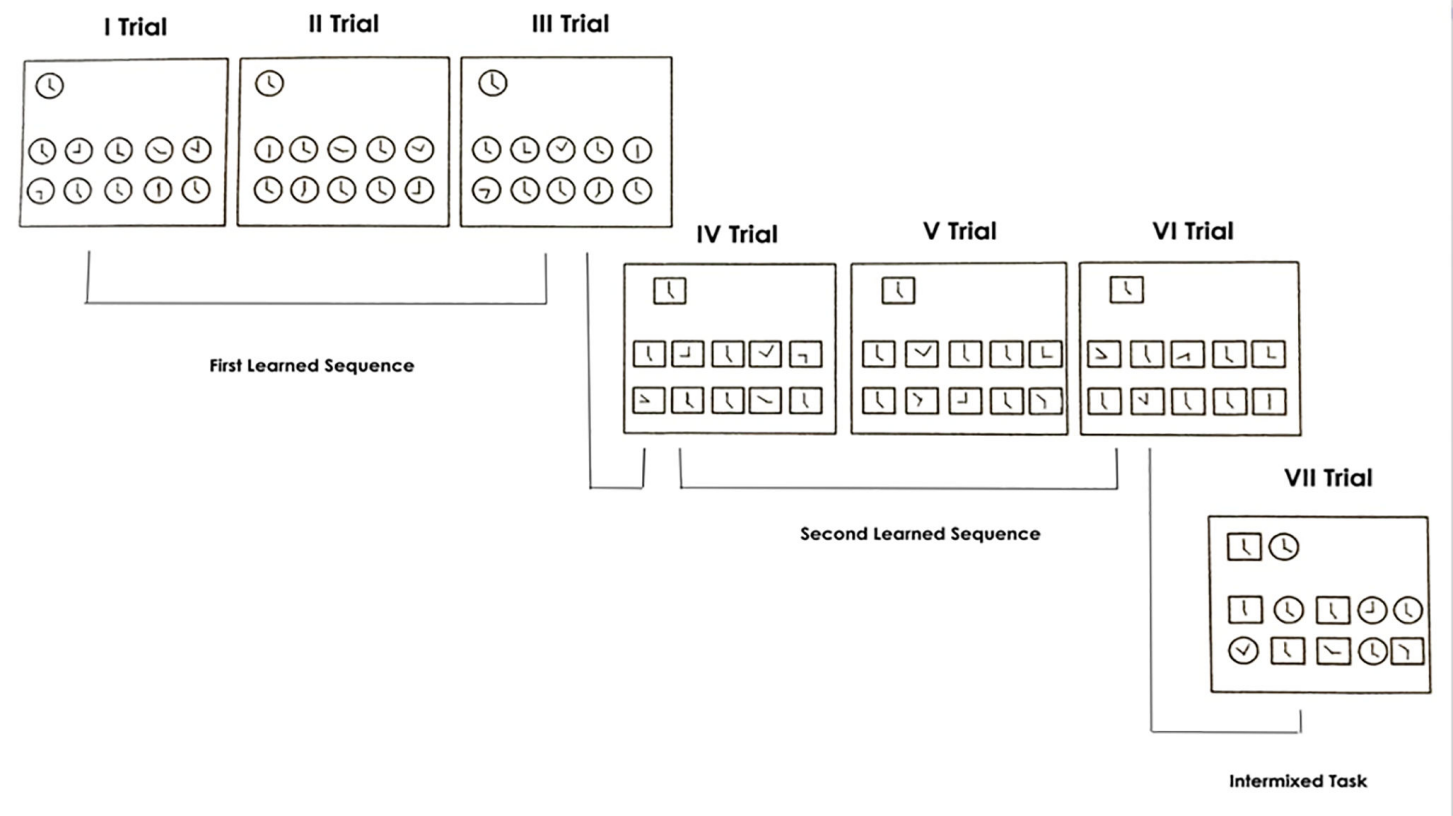
Condition with contextual cue.

In the condition with contextual cue, the shape of the clock-face changed from the fourth trial onwards (see Figure 1). More precisely, in the first, second and third trial, the clock shape was a circle, in the fourth, fifth and sixth trial it was a square; whereas in the seventh trial the circle and square shapes were intermixed.

**Figure 2:**
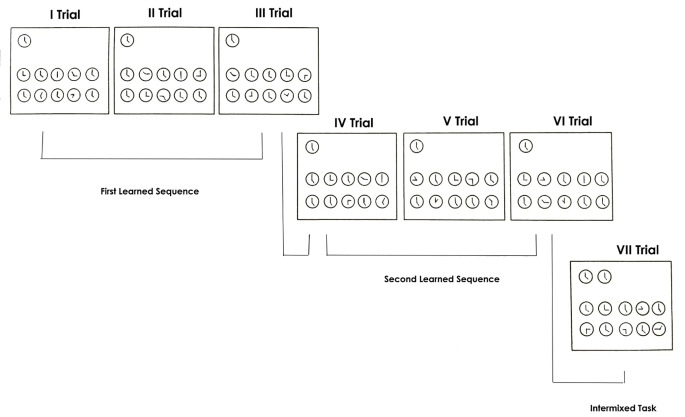
Condition without contextual cue.

In the condition without contextual cue, the context was always the same, that is, all the clock-faces had the shape of a circle on all the sequence tasks (see Figure 2).

The two conditions were randomized. The number of the two targets was equal to that of the previous condition. The loss of the processing speed between the two conditions was considered as an index of the rigidity/flexibility continuous process. In particular, we were interested in the loss of processing speed in the shift from third to fourth trial, and from sixth to seventh trial in the two conditions.

### Measurements

The subject’s performance was coded as follows:

- The number of correct responses—CR (since the time is fixed, a period of 2 minutes of continuous attention for each trial, the number of correct responses is considered as an index of processing speed); as many works suggest, processing speed measures automaticity and fluency of cognitive performance across a wide variety of tasks (Bryan & Luszcz, 2001; Finkel, Reynolds, McArdle, & Pederson, 2005; Kail & Salthouse, 1994; Salthouse, 2005).
- The number of false alarms—FA (erroneous “detection”) that measures the accuracy of performance.

### Statical Analyses

Data were analyzed using SPSS 14.0 for Windows. The descriptive statistics of the dependent variables were tabulated and examined. Alpha-level was set at .05 for all statistical tests. In the case of significant effects, the effect size of the test was reported. The effect sizes were computed and categorized according to Cohen (1988). The Greenhouse-Geisser adjustment for nonsphericity was applied to probability values for repeated measures.

## Results

Results are presented, as mentioned in the procedure section, with reference to 4 units: 1) *first learned sequence*, with the first three trials indicating the 4 target, to detect automaticity of the first target; 2) *second learned sequence*, with the second three trials indicating the five target, to detect the automaticity of the second target; 3) *Target shift* (from 4 to 5 o’clock, from 3rd to 4th trial), to detect the rigidity/flexibility due to the shift; 4) *Intermixed task* (7th trial) ), to detect the rigidity/flexibility due to the the intermixed stimuli.

### First Learned Sequence (1^st^, 2^nd^, 3^rd^ Trials)

Table 1 shows the means and standard deviations of CR related to the 4 o’clock target. Table 2 shows the means and standard deviations of FA related to the 4 o’clock target. A repeated measures analysis of variance was carried out with two within subject factors: 2 (contextual features: with vs. without contextual cue) X 3 (Sequences: first, second and third trial). Since the first three trails in the conditions with and without contextual cue were the same, we awaited no differences related to contextual features.

**Table 1:** Means (M) and Standard Deviations (SD) of CR Related to the Targets 4 and 5 Hours in All Trials

Trials	Target	With Contextual-Cue Condition		Without Contextual-Cue Condition	
		*M*	*SD*	*M*	*SD*
I	4	27.50	4.36	27.90	6.31
II	4	31.80	3.80	31.90	4.82
III	4	35.60	3.46	34.80	4.09
IV	5	35.50	2.83	27.43	6.14
V	5	36.83	2.15	30.69	4.50
VI	5	37.91	1.53	33.90	3.56
VII	4	35.85	3.85	30.26	5.47
	5	36.91	3.96	29.77	5.39

**Table 2:** Means (M) and Standard Deviations (SD) of FA Related to the Targets 4 and 5 Hours in All Trials

Trials	Target	With Contextual-Cue Condition		Without Contextual-Cue Condition	
		*M*	*SD*	*M*	*SD*
I	4	14.69	6.99	16.95	7.55
II	4	10.32	5.35	11.92	5.80
III	4	6.86	4.26	8.79	5.72
IV	5	10.74	4.95	13.58	6.86
V	5	8.88	5.15	9.62	4.65
VI	5	6.25	4.00	6.43	3.76
VII	4	6.23	2.50	9.82	3.55
	5	6.22	4.11	9.63	3.45

**Figure 3:**
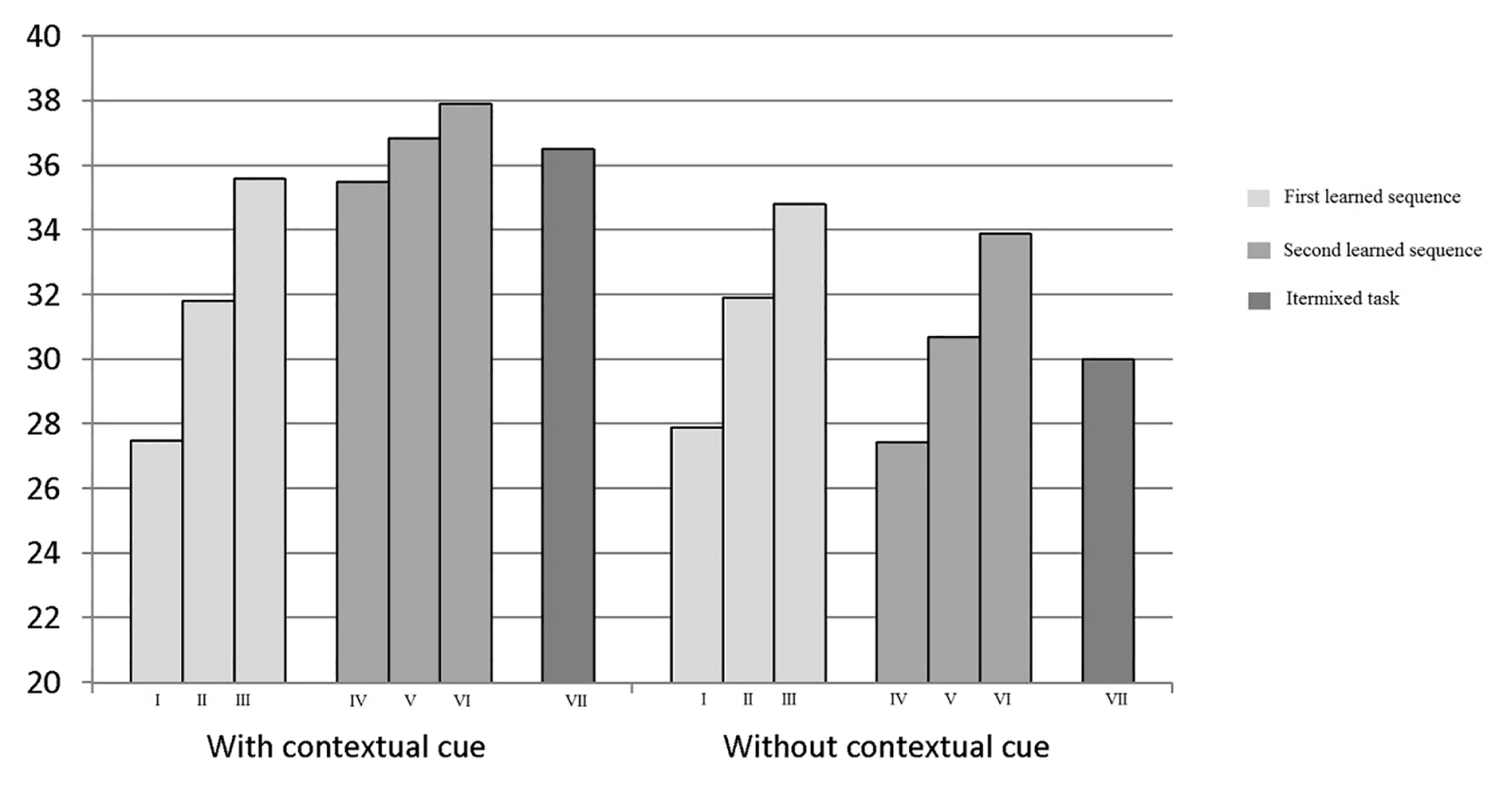
CR related to the targets 4 and 5 hours in all trials.

**Figure 4:**
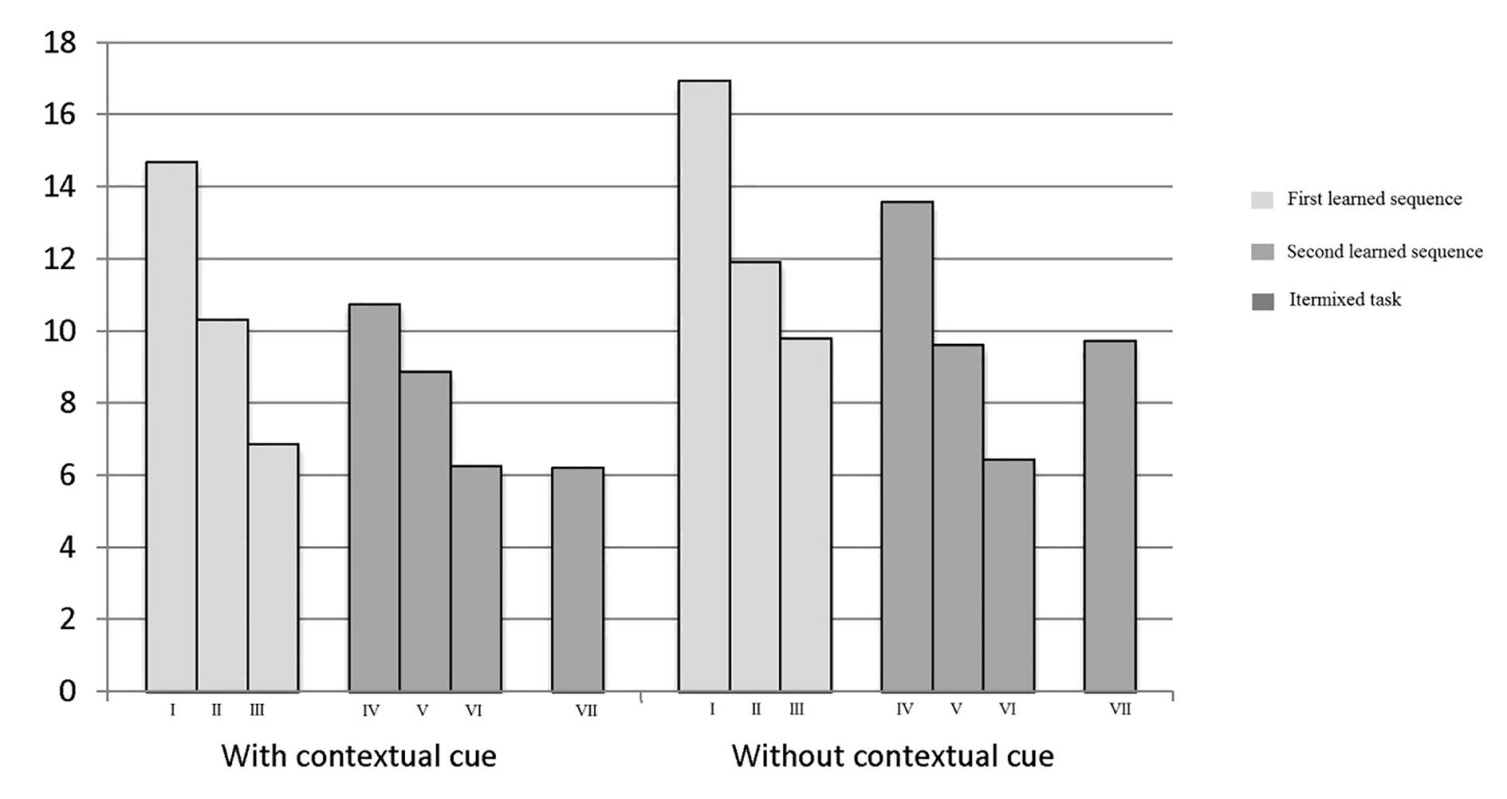
FA related to the targets 4 and 5 hours in all trials.

With reference to both CR and FA, the “sequences” factor showed significant effects, respectively *F*(2, 238) = 199.45, *p* < .0001, *d* = 0.90 and *F*(2,238) = 298.21, *p* < .0001, *d* = 0.90. This result indicated that when subjects identified the same target stimulus in many trials the speed of execution and accuracy (decrease in the number of errors) increased, respectively, from 27.50 and 27.90 to 35.60 and 34.80 and from 16.95 and 14.69 to 8.79 and 6.886 (Figures 3 and 4).

### Second Learned Sequence (4^th^, 5^th^, 6^th^ Trials)

As mentioned above, CR and FA of the fourth, fifth and sixth trials are presented in Tables 1 and 2. A repeated measures analysis of variance was carried out with two within subject factors: 2 (contextual features: with vs. without contextual cue) X 3 (Sequences: fourth, fifth and sixth trials).

With reference to both CR and FA, the “sequences” factor showed significant effects, respectively *F*(2, 238) = 218.56, *p* < .0001, *d* = 0.90 and *F*(2, 238) = 78.45, *p* < .0001, *d* = 0.81. This result indicated that the subjects automated the new target both in condition with and without contextual cue, from 35.50 and 27.43 to 37. 91 and 33.90 and from 13.58 and 10.74 to 6.43 and 6.25. Similarly, in this sequence task, the contextual cue was added as a counterbalancing condition, and, as expected, it had no statistical effect.

### Target Shift (from 4 to 5 o’clock, from 3^rd^ to 4^th^ Trials)

CR of the third and fourth trials are presented in Table 1. To examine the role of contextual features, *t*-test for dependent measures was carried out. We compared the performance in trials in which the target clock-face was shifted (3^rd^ and 4^th^ trials), in both conditions. Results showed no significant effect in the condition with the contextual cue, *t*(119) = 0,34; *p* = .61. A significant effect in the condition without contextual cue was found, *t*(119) = 4.41, *p* < .05. As expected, without contextual cue, the subjects showed a loss of processing speed in the trial in which the target changed. Whereas the subjects did not show a loss of processing speed in the trials with contextual cue. These results evidenced the role of contextual features in flexibility.

### Intermixed Task (7^th^ Trial)

CR of the seventh trial is presented in Table 1. To examine the role of contextual features, *t*-test for dependent measures was carried out. We compared the performance between the sixth and seventh trials in both conditions. Results showed no significant effect in the condition with contextual cue for the two clock-face targets, *t*(119) = 0.16; *p* = .81 and *t*(119) = 0,02, *p* < .62. As expected, a significant effect in the condition without contextual cue for both target 4 and 5 was found, respectively *t*(119) = 8.23, *p* < .03, and *t*(119) = 6.26, *p* < .05. Similarly, in the intermixed task, the participants showed a loss of processing speed without contextual cue.

## Discussion

The specific aim of the present study was to demonstrate that participants can increase processing speed (automatization) of two sequence tasks, and that such increment can be tied to contextual features associated with the two tasks. The first goal was to induce an automatization process of two sequence tasks. Secondly, we examined whether contextual features influenced flexibility on the learned sequences.

As expected, the results showed that when subjects identified the same target stimulus on the first and second learned sequence, the speed of execution and the accuracy (decrease in the number of errors) increased. Therefore, performance improved through exercise, and, the two sequence tasks were learned progressively. These results are in line with the classical theories of skill acquisition (Fitts & Posner, 1967) for which the performance becomes automatized, and there may be an increment of speed and decrement of errors, as a function of practice (D’angelo, 2012, 2013, 2014; Ruitenberg et al., 2012a, 2012b, 2015; Ruitenberg, Abrahamse, & Verwey, 2013).

With reference to the second goal, results indicated that contextual features influenced flexibility on the learned sequences. More precisely, the target shift (unexpected change of the stimulus to detect), which requires reactivation of the controlled processes, produced a loss of processing speed in trials without contextual-cue, whereas, in the condition with contextual-cue the subjects did not show loss of processing speed. Furthermore, we also found loss of processing speed when the two targets were randomly intermixed in the without contextual-cue condition compared to the condition with contextual-cue. Thus, it seems plausible to assume that contextual features play an important role in automatization processes. The difference between the performance in the target shift and in the intermixed task reveals that the benefits of automation are higher and more significant when the subject was presented the contextual-cue. These results are in line with literature on context-specific proportion congruent effects showing evidence for flexible transfer of context-specific control across different items (Bugg & Crump, 2012; Crump, Brosowsky, & Milliken, 2016; Crump & Logan, 2010; Crump & Milliken, 2009; Crump, Milliken, Leboe-Mcgowan, Leboe-Mcgowan, & Gao, 2018).

It is interesting to note that when participants identified the same target on the first and second learned sequence, the contextual cue had no effects. These two sequences implied activation of the automatic processes. In addition, the process became more automatic after the first learned sequence, and the controlled processes were again needed at the start of the second learned sequence. Instead, when the subjects simultaneously detected two targets in the intermixed task, the contextual cue showed a significant effect. This task required the activation of both controlled and automatic processes. Thus, all the results indicate that participants can switch between both processes and that context cues facilitate this switching. Therefore, it is reasonable to assume that contextual features facilitate the activation of controlled and automatic processes and that a learned task can become more flexible through the inclusion of context-specific features.

In light of the above, the present study aim to fill the knowledge gaps about the conditions under which the contextual cue is involved in the automatic processing. In particular, with reference to previous studies (D’Angelo, 2012, 2013, 2014), this work is based on some substantial methodological differences which could explain the different results. The design of this experiment doesn’t include a practice phase, because this can facilitate the rapid learning of the two sequence tasks. The intermixed phase of the circles is controlled by the two base-line sequences of the first and second learned circle sequences. Since both sequences are base-line controlled, it is reasonable to assume that the only change between the conditions with and without contextual cue is due to the facilitation of contextual features.

Moreover, D’Angelo et al. (2012, 2013, 2014) employed a localization task in which two sequences were used to assign the location of the target on every trial; the location of two targets was determined by two complementary sequences that were derived from the artificial grammars used in their previous research. This localization task consisted of two main phases, a training phase and a transfer phase. While, in this study, we used a visual search task with two learned sequences phases and one intermixed phase. Given the different results between our work and previous studies, probably, the type of task can reflect the absence or presence of reliance on contextual features.

Other previous studies investigated the conditions that promote feature search (Bacon & Egeth, 1994; Leber & Egeth, 2006a, 2006b; Leonard & Egeth, 2008). More specifically, Graves and Egeth (2015) analyzed the conditions under which the resistance to attentional capture can be found. They employed a visual search task in which the shape of the target was a circle with other shapes as distractor stimuli (diamond, square and triangle). They performed three experiments to examine the conditions in which the effect of color uncertainty of shape stimuli can be observed. The results of Experiment 1 indicated that attentional capture occurs when the learning of an association between the shape and a particular color or colors was prevented. Whereas, in Experiments 2 and 3, the attentional capture did not occur even when most color or singleton color had two possible values. Probably, in these experiments the color of the shapes was an unable contextual cue to determine an automatic processing. Anyway, it is possible that other factors could explain the interplay among contextual cue learning, visual attention and task switching, such as: the type of shape and the experience with the salient feature of the distractor. Future works could test this hypothesis.

In short, we assume that the effects of context are a consequence of the experimental design used. Future research is required to support for this view. Anyway, the current study introduces significant novelty about the methodological strategies under which is possible to analyze the effect of contextual features on automatic processing of two learned sequences.

From a conceptual point of view, the present study suggests that the classical distinction between automatic and controlled processing as separable processes can be softened, as the automatic processes can gradually become flexible and context-dependent, as shown in this work. Also, our findings have a theoretical implication; they highlight the necessity to reconsider the classical theories and support the refined theories of automaticity, which assume more flexibility and adaptability of automatic processing (Fabio, 2017; Fabio, Caprì, Buzzai, Pittalà, & Gangemi, 2019; Kiefer, 2007, 2012; Kiefer & Martens, 2010; Moors & DeHouwer, 2006; Naccache et al., 2002; Neumann, 1990). From the prospective of refined theories, the flexibility reported in this study can be explained as the result of reliance on two contextual-specific features, which are incorporated in the task. The inclusion of these context features can in turn lead to context-specific expression of the learned task when those contextual features are presented and are able to effectively cue the retrieval of appropriate knowledge.

In conclusion, the present study highlights two key points: the first concerns the possibility that context-specific features can affect the learned task and can implement flexibility in automatic processing; and the second concerns the revision of theories on automaticity in the direction of the complex model in which automatic and controlled processes are conceptualized as two continuous processes which interact with each other. It is instructive to look at the role of contextual features in a cognitive task from a complex prospective, by recognizing that human performance often results from an interplay between automatic and controlled processing and these processes may be mediated by systems that evolve to satisfy the need for operation in a complex environment, where attention must be guided to process selectively critical stimuli.

### Compliance with Ethical Standards

The authors declare that they have no conflict of interest and no funding. All procedures performed in studies involving human participants were in accordance with the ethical standards of the institutional and/or national research committee and with the 1964 Helsinki declaration and its later amendments or comparable ethical standards. Informed consent was obtained from all individual participants included in the study.
